# Supplementary material for: How sturdy is your memory palace? Reliable room representations predict subsequent reinstatement of placed objects
Source: bioRxiv. 2024 Nov 26:2024.11.26.625465. Preprint. [Version 1] doi: 10.1101/2024.11.26.625465 (PMC11623609; doi:10.1101/2024.11.26.625465)
Supplement: Supplementary file 1 [file NIHPP2024.11.26.625465v1-supplement-1.pdf]

bioRxiv preprint doi: https://doi.org/10.1101/2024.11.26.625465; this version posted November 26, 2024. The copyright holder for this preprint (which was not certified by peer review) is the author/funder, who has granted bioRxiv a license to display the preprint in perpetuity. It is made available under aCC-BY 4.0 International license.

7 Supplementary Information

Supplementary Table 1 Virtual memory palace room music

Room id	Room Name	Track Name	Track Artist
1	Antiques Room	Rio De Colores	Strunz & Farah
2	TV Room	Birds	Rolando Masís-Obando
3	Candy Room	Liar Liar	The Castaways
4	Classroom	Scatting at the 18th Grammy Awards, Feb. 1976	Ella Fitzgerald & Mel Tormé
5	Tool Room	brazil loops 1	motion_correct
6	Bedroom	Rero	Burne Holiday
7	Floating Islands Room	Brooks Was Here (800% slower) (Rolando Masís-Obando StretchMix)	Thomas Newman
8	Planet Room	Electric à la Mass Effect	Rolando Masís-Obando
9	Computer Store Room	Inmersión	Éditus 360
10	Painting Room	Juana y Mi Hermana	Sonámbulo Psicotropical
11	Storage Boxes Room	Aragorn's Speech at the Black Gate	from Lord of the Rings
12	Chess Room	Prelude in G Minor (Op. 23 No. 5)	Sergei Rachmaninoff
13	Empty Room	Wrong Side	Strapping Young Lad
14	Cat Portraits Room	Symphony No.8 "Unfinished" D 759	Franz Schubert
15	Ruins Room	Memory (Extended)	Nathan Prillaman
16	Clocks Room	Birds Stretched	Rolando Masís-Obando
17	Crystals Room	colorthought - crash _JR 11.4.16	Jakob Reske
18	Colorful Wall Room	Zaggazaggа	Rolando Masís-Obando
19	Altar Room	Throatsinging	Rolando Masís-Obando
20	Applecreates Room	Tron Overture (form the Motion Picture TRON: Legacy)	Daft Punk
21	Birthday Party Room	Birthday Horror	Rolando Masís-Obando & the Ken Norman Lab
22	Firepit Room	fire crackles	N/A
23	Human Portraits Room	What Way?	Javier Masís

bioRxiv preprint doi: https://doi.org/10.1101/2024.11.26.625465; this version posted November 26, 2024. The copyright holder for this preprint (which was not certified by peer review) is the author/funder, who has granted bioRxiv a license to display the preprint in perpetuity. It is made available under aCC-BY 4.0 International license.

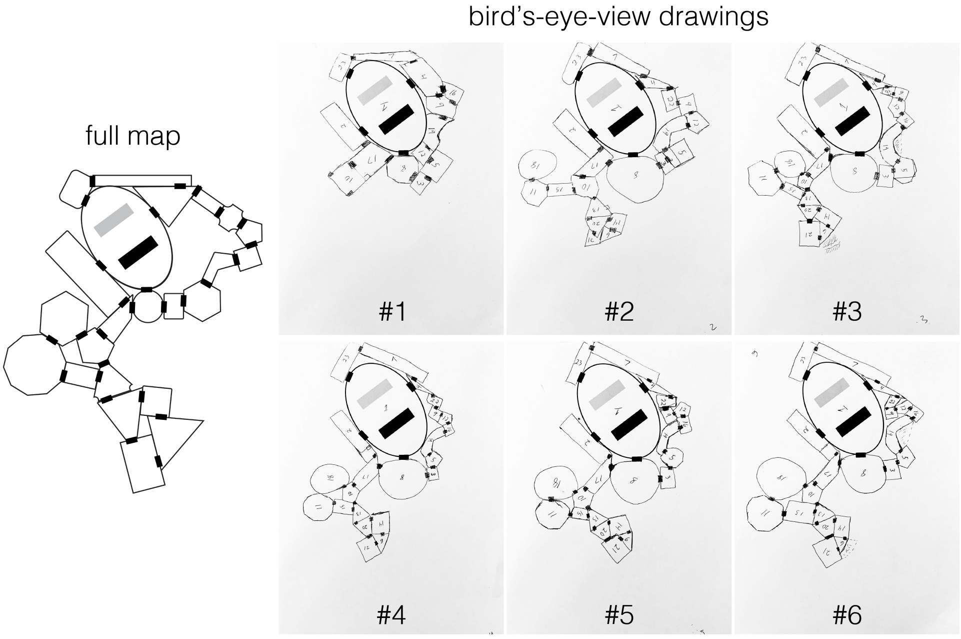

Figure 1–Figure supplement 1. Example bird's-eye-view drawings from a single selected participant. Each participant was asked to draw multiple bird's-eye-view maps of the environment across pre- and post-learning sessions to monitor training progress. Each participant was given two sheets of paper, one for the map they had to draw with a central room drawn in, and another with a legend containing the room name and pre-determined room number to facilitate map drawing and room labeling.

bioRxiv preprint doi: https://doi.org/10.1101/2024.11.26.625465; this version posted November 26, 2024. The copyright holder for this preprint (which was not certified by peer review) is the author/funder, who has granted bioRxiv a license to display the preprint in perpetuity. It is made available under aCC-BY 4.0 International license.

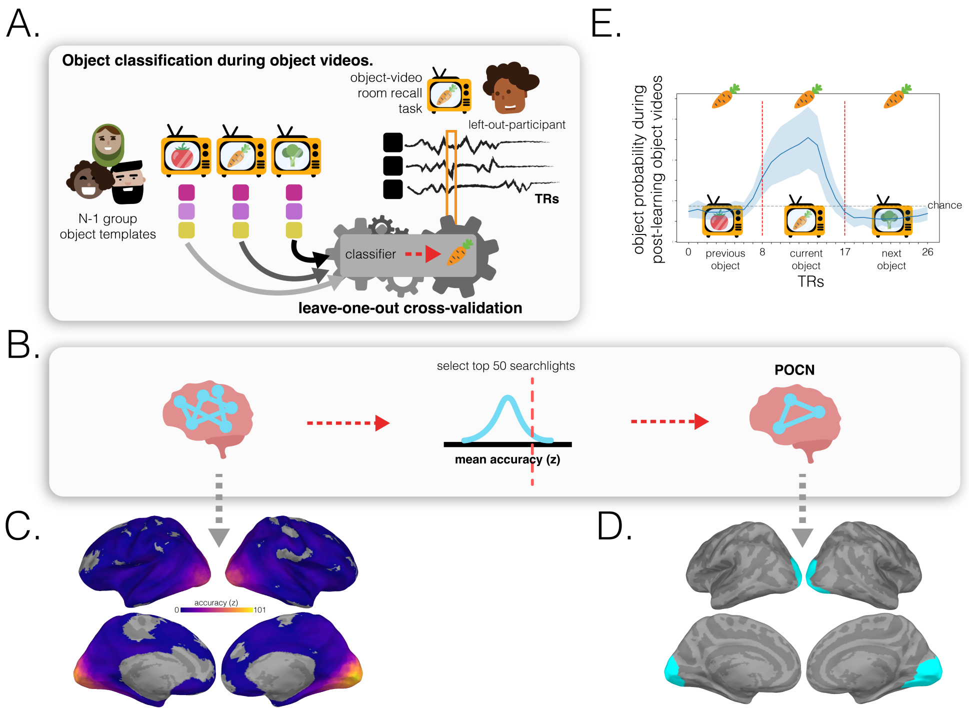

Figure 3–Figure supplement 1. Perceived object classifier network (POCN) methodology and surface maps. (A.) As for the ROCN, the characteristic object patterns of the N-1 group were used to train a multinomial logistic classifier. Here, this classifier was applied to timepoints when the left-out participant was viewing objects during the post-learning object videos, e.g. viewing a video of a carrot in this example. **(B.)** After object classification was performed for both post-learning object video runs for each participant, average classification accuracies across participants for each searchlight were then averaged across both runs and then z-scored relative to a null distribution. The top 50 searchlights were then selected to form the POCN. **(C.)** Average object classification accuracy during object encoding (thresholded to show searchlights with above-chance accuracy). **(D.)** Perceived Object Classifier Network. The surface map shows the top 50 best object classifying searchlights during both post-learning object video tasks across participants. **(E.)** Average object classifier evidence for the object that was currently being viewed, averaged within the POCN; evidence for this same object is also shown for earlier timepoints when another object was being viewed and for timepoints when the next object in the trial was being viewed. Classifier evidence for the object is above chance only when the participant is viewing the video of the matching object. Error bands show standard deviation across participants.

bioRxiv preprint doi: https://doi.org/10.1101/2024.11.26.625465; this version posted November 26, 2024. The copyright holder for this preprint (which was not certified by peer review) is the author/funder, who has granted bioRxiv a license to display the preprint in perpetuity. It is made available under aCC-BY 4.0 International license.

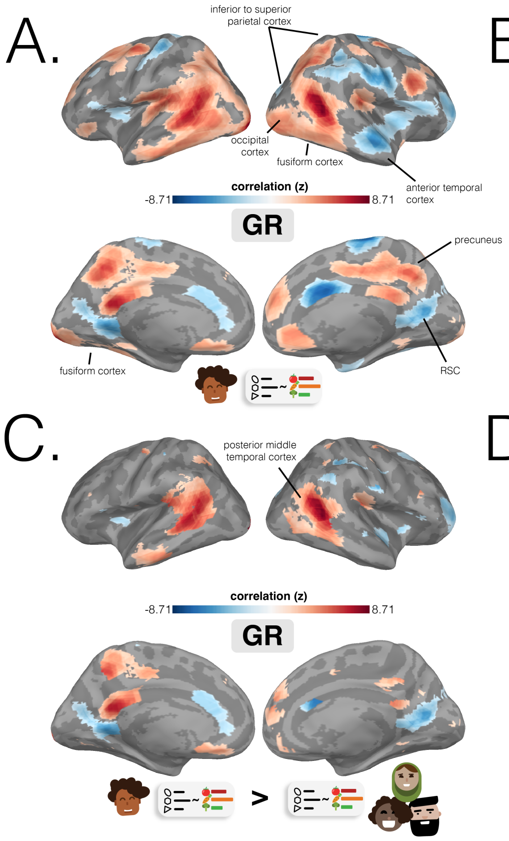

Figure 5–Figure supplement 1. Predicting POCN object reinstatement from room reliability. (A.) Regions where room reliability predicted POCN object reinstatement in guided recalls. **(B.)** Regions where room reliability predicted POCN object reinstatement in free recalls. **(C.)** Regions where room reliability predicted POCN object reinstatement in guided recalls and there was also a predictive benefit from participant-specific room reliability. **(D.)** Regions where room reliability predicted POCN object reinstatement in free recalls and there was also a predictive benefit from participant-specific room reliability. The surface maps presented in **A** and **B** were statistically thresholded by comparing correlations to a null distribution and then FDR-correcting for $q < 0.05$. The surface maps presented in **C** and **D** show the intersection of the participant-specific models (**A** and **B**) and the regions where there was a significant positive difference in the coefficient of determination between the original participant-specific model and the N-1 group model. Statistical significance for the differences between the coefficients of determination was determined by comparing the differences to a null distribution and FDR-correcting for $q < 0.05$. All four surface maps are colored based on the magnitude of the z-scored correlation values of the participant-specific model, with blue showing negative and red showing positive relationships, respectively.
